# Millennial scale maximum intensities of typhoon and storm wave in the northwestern Pacific Ocean inferred from storm deposited reef boulders

**DOI:** 10.1038/s41598-020-64100-6

**Published:** 2020-04-29

**Authors:** Kenta Minamidate, Kazuhisa Goto, Masashi Watanabe, Volker Roeber, Ken Toguchi, Masami Sannoh, Yosuke Nakashima, Hironobu Kan

**Affiliations:** 1 Graduate school of Science, Tohoku University, Aoba 468-1 E303, Aramaki, Aoba-ku Sendai, 980-0845 Japan; 2 International Research Institute of Disaster Science, Tohoku University, Aoba 468-1 Aramaki, Aoba-ku, Sendai 980-0845 Japan; 3 Present Address: Department of Earth and Planetary Science, The University of Tokyo, 7-3-1 Hongo, Tokyo, 113-0033 Japan; 4 School of Engineering, Tohoku University, Aoba 468-1 E305, Aramaki, Aoba-ku, Sendai 980-0845 Japan; 5 Present Address: Faculty of Science and Engineering, Chuo University, 1-13-27 Kasuga, Bunkyo-ku, Tokyo, 112-8551 Japan; 6 Univ Pau & Pays Adour/E2S UPPA, Chaire HPC-Waves, Laboratoire des Sciences de l’Ingénieur Appliquées à la Méchanique et au Génie Electrique-Fédération IPRA, EA4581, 64600 Anglet, France; 7 University of the Ryukyus, 207 Uehara, Nishiharasho, Nakagami, Okinawa, 903-0213 Japan; 8 Mikuniya Corporation, 2-9-3 Sannou, Hakata-ku, Fukuoka, 812-0015 Japan; 9 NIT, Ariake College, 150 Higashihagio-Machi, Omuta, Fukuoka, 836-8585 Japan; 10 Graduate School of Integrated Sciences for Global Society, Kyushu University, 744 Motooka, Nishi-ku, Fukuoka, Japan

**Keywords:** Natural hazards, Ocean sciences, Climate sciences, Palaeoceanography, Palaeoclimate

## Abstract

Typhoons and associated storm waves in the northwestern Pacific Ocean commonly cause coastal disasters. The possibility remains that an even stronger typhoon than the strongest one observed to date might have occurred before. The development of a method to estimate a maximum intensity of past typhoons over thousands of years is important for paleoclimatology, paleoceanography and disaster prevention. Numerous storm wave boulders exist on reefs in the Ryukyu Islands, Japan, which have been deposited to their present position by the cumulative effects of the past storm waves. These boulders can be used as proxies for the hydrodynamic conditions of the largest waves from past events. Here, we present numerical computations for storm waves and boulder transport with the boulder distribution as a constraint factor to estimate the maximum intensities of storm waves and their causative typhoon events over the past 3500 years. Though the intensities of the maximum estimated waves and associated typhoon events were slightly stronger than those recorded over the past ~70 years in the Ryukyu Islands, our results suggest that no abnormally intense typhoon has struck the Ryukyu Islands in the past 3500 years. The potential impact from tsunamis remains uncertain; however, our results are meteorologically reasonable.

## Introduction

Tropical cyclones and associated storm waves are extreme ocean phenomena that can lead to significant geomorphic changes and serious disasters in the coastal zone^1,2^. Especially, the northwestern Pacific Ocean is one of the most active tropical cyclone areas in the world^3^, and thus it is necessary to take measures against the impact of intense typhoons in this area. In Japan, records of typhoon measurements have been available since A.D.1951^4^. While measurement records in Japan are relatively long (~70 years) compared to other countries, the record period is still too short for an accurate assessment of long-term risks. It is therefore difficult to exclude the possibility that extremely intense typhoons and consequent wave impacts have been generated in historical and prehistoric times in the northwestern Pacific Ocean. Although historical or archeological records are indeed useful, their availability is sometimes limited depending on both  location and history^5,6^. Also, intensities of typhoons are difficult to be estimated from these records. Geological records are crucial to perform the necessary risk assessment of storm surges and waves along the coast over a few hundred to thousand years before the measurement records^7–10^. Although there are few previous studies^11,12^, the methodology to reconstruct past wave conditions and the causative typhoon intensities from the geological records are still limited.

**Figure 1:**
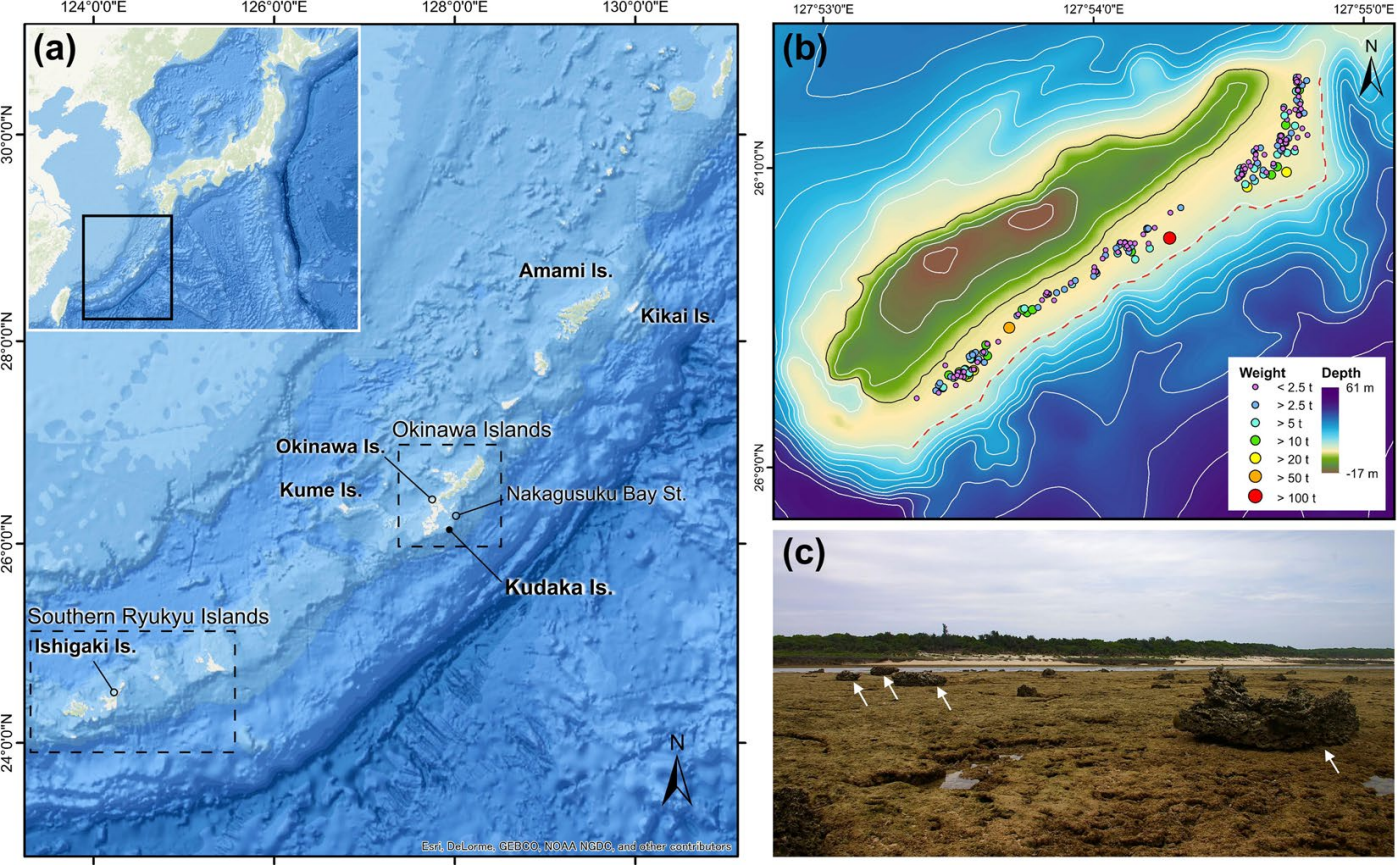
(**a**) A map of Ryukyu Islands. Kudaka Island is shown as a black solid circle. A base map is provided by ESRI Japan. (**b**) A map showing the boulder distribution in Kudaka Island (after Goto *et al*.^16^). Red dotted line depicts the reef edge. A base map is created using data provided by Okinawa Prefecture. (**c**) A photo of the boulders on the reef. The photo was taken from the reef crest toward the land.

Large coastal boulders in excess of 1 m in dimeter are one of the useful sedimentary records to reconstruct frequency and intensity of past extreme wave events^11–15^. Here we conducted a field survey and numerical modeling efforts at Kudaka Island, Japan (Fig. 1), where numerous storm wave boulders are reported^16^, in order to estimate the maximum intensities of the storm waves and the associated typhoon events over a few thousand years around the Ryukyu Islands.

## The Kudaka boulders

Numerous boulders are deposited on the reefs along the Ryukyu Islands and discrimination of their origin has been an important issue during past 30 years^17,18^ because the islands are situated in the path of strong typhoons as well as the Ryukyu Trench, which may induce large earthquakes and tsunamis.

Goto *et al*.^18^ suggested that there is a clear difference in the spatial and clast size distributions of the boulders deposited by storm waves and tsunamis in the Ryukyu Islands. Indeed, the storm wave boulders are distributed throughout the Pacific coasts of Ryukyu Islands with limited distance from the reef edge (~300 m) because the storm boulders accumulate in accordance with the attenuation of the storm waves^18^. The boulders on the Kudaka Island show a typical distribution of storm boulders so that they are interpreted as stemming from storms^16^.

Whether the Ryukyu Trench is coupled or decoupled is still an ongoing debate^19,20^. However, no large historical earthquakes were reported except for some moderately large ones with magnitudes of around 8.0^21,22^. The 1771 Meiwa earthquake and tsunami that occurred off the Ishigaki Island (Fig. 1) was the only known event that generated a large tsunami with about 30 m in run-up height^23^. The tsunami might have been generated by an earthquake with Mw = 8.0^24^, with possible contributions of a submarine landslide^13,23,25^. Indeed, the affected area by the 1771 tsunami is limited only to the southern Ryukyu Islands and no influence was recorded at the Okinawa Islands including the Kudaka Island^26^. On the reefs of these tsunami-affected southern islands, extremely large boulders are deposited along the coastline over the ~1.5 km wide reef, with a distinctly different distribution from the storm boulders^26^. Hence, these boulders can be of tsunami origin. These sedimentological criteria for the differentiation was supported by numerical modeling by Watanabe *et al*.^27^; they suggested that boulders near the reef edge can be explained by the normal size of storm wave while those along the coastline at southern islands should have been deposited by tsunamis.

It is obvious that the boulders along the Ryukyu Islands can be characterized by the preferential distribution: tsunami boulders are observed only on the southern islands but not at central to northern Ryukyu Islands including Kudaka Island^18^. In this point, the Ryukyu Islands is a rare place where discrimination of tsunami and storm boulders were successfully performed.

Although the boulders at Kudaka Island are interpreted to stem from storm origin^16^, can we fully exclude the potential influence of tsunamis? A Tsunami is an event that can significantly disturb the distribution of storm wave boulders^18,26,27^. If large tsunami occurred in the past near the Kudaka Island, boulders cannot remain along the reef edge and all of them should have been moved landward significantly as is observed in the southern islands where was affected by the 1771 Meiwa tsunami. However, there is no evidence that boulders at Kudaka Island moved beyond the distribution limit of storm wave boulders^16^ so that we can exclude the possible influence of large tsunamis with wave force to change the boulders’ distribution significantly. While, we cannot fully exclude the possibility that small tsunamis might have occurred in the past around the Kudaka Island that were too weak to have changed the distribution of storm boulders (Fig. S1). However, even such small tsunamis occurred in the past, they do not affect to the following discussion about the estimation of typhoon intensities (Fig. S1).

Reef boulders on the coral reef flat of Kudaka Island, were originally composed of reef material from the reef edge. They are commonly dislocated by waves and it is well known that their clast size and spatial distributions are strongly controlled by the wave deformation due to the reef topography^16^. Storm waves attenuate on the shallow and flat reef^28,29^, thus they cannot maintain the wave force to move boulders a long distance on the reef. Numerous small storm waves have potentially be generated by small but frequent typhoons. Such small waves probably have a role to emplace small boulders and slightly move them landward. However, since smaller waves attenuated faster, they can only transport boulders in a shorter distance (Fig. S1). On the other hand, larger waves can overwrite the previous clast size and spatial distribution of boulders more landward. Hence, storm wave boulders are distributed over a limited distance from their sources (=reef edge) unless they were transported by stronger wave forces.

According to the field observation and satellite image analyses, new storm wave boulders have frequently been emplaced and changed their position on the reef after the large typhoon events even in the recent years^16^. Considering this fact, storm wave boulders were probably shifted landward to their present positions by the intermittent but cumulative effect of numerous waves generated by numerous typhoon events. Thus, it is possible to assume that the present distribution of storm wave boulders, especially certain weight of boulders deposited in the maximum landward distances from the reef edge, could have been formed by the maximum wave since their deposition on the reef of about few thousand years ago^16^. This in turn suggests that the maximum storm wave can be estimated using the present distribution of storm wave boulders.

## Cross sectional calculation of storm wave and boulder transport

By assuming mean sea level, the calculation of boulder transport with 133 cases of initial wave condition were performed (Table S1). Although the offshore waveform is almost constant regardless of time, the waves shorten in wavelength and increase in height as they go to shallow sea (Fig. S2). The wave height further grows near the reef edge before it breaks. The water level became the highest at offshore of the reef edge (Fig. S3), and then it dropped quickly as the water depth became shallower on the reef slope. The water level remarkably dropped on the reef flat, and finally the maximum water level on the reef decreased to about one third of offshore level. Similarly, the maximum water velocity increased drastically at offshore of the reef edge and then water velocity suddenly decreased on the reef, although a small peak of velocity was observed at around the outer slope of the shallow lagoon. Under such wave condition, boulders are intermittently moved landward by number of waves and then stopped at the places where resisting forces of each boulder become equal to the wave forces (Fig. S4).

## Typhoon and wave field

**Figure 2:**
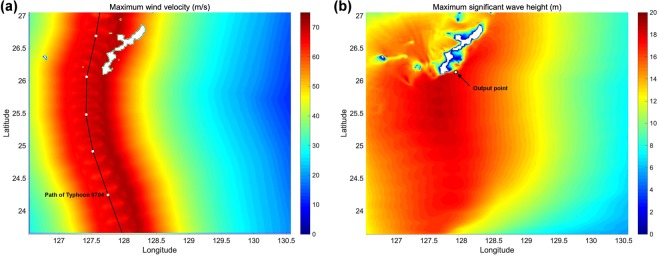
Results of storm simulation for caseT01. (**a**) Maximum wind velocity (m/s), and (**b**) maximum significant wave height (m). See Fig. S5 for other cases.

**Figure 3:**
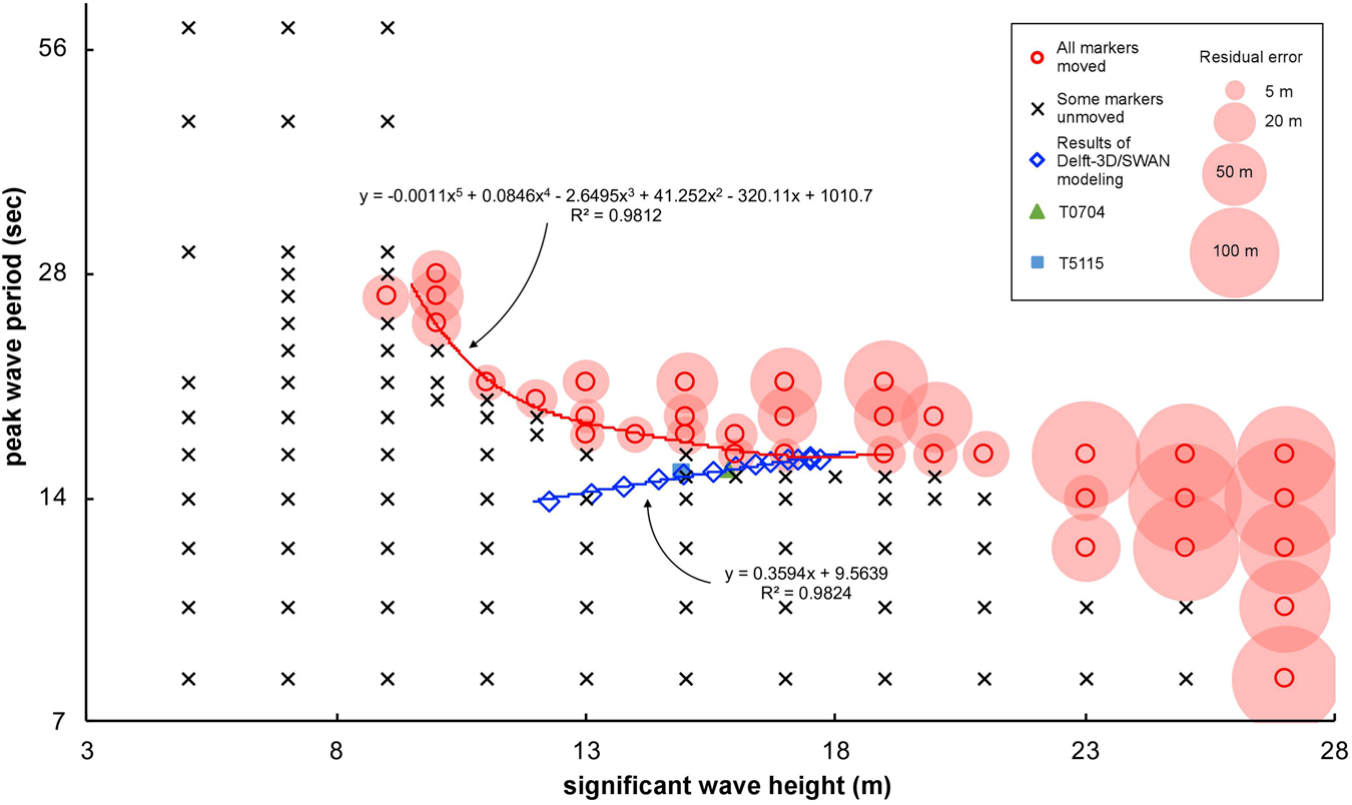
The results of wave and boulder transport calculations by BOSZ, and storm simulation by Delft-3D/SWAN. Red circle: wave condition that can move all marker boulders. A red regression curve is drawn using red plots with the smallest peak wave period in each significant wave height ranging from 10 to 19 m. Cross: wave condition that cannot move one or more marker boulders. Blue diamond: results of storm wave calculated by Delft-3D/SWAN modeling.

The simulation of typhoon and consequent wave field were conducted in 17 cases (Fig. 2). The strength of wave distribution mainly corresponded to the wind speed distribution. Furthermore, the maximum significant wave height (H_s_) was the highest on the southern side of Okinawa Island where Kudaka Island is located. Results showed that H_s_ and peak wave period (T_p_) also become larger as the strength of the typhoon becomes stronger (Table 1, Fig. 3). In the calculation of typhoon 0704’s hindcasting, H_s_ at Nakagusuku Bay was calculated to be 13.95 m. This is consistent with the actual observed record of 13.61 m^30^, implying that our simulation was valid.

**Table 1:** Storm conditions (central pressure and maximum wind speed) and output of storm simulation (significant wave height and peak wave period).

	P (hPa)	V (knots)	H_s_ (m)	T_p_ (sec)
caseT01	880	145	17.51	15.76
caseT02	885	140	17.69	15.77
caseT03	890	135	17.49	15.83
caseT04	895	130	17.48	15.78
caseT05	900	125	17.23	15.74
caseT06	905	120	17.04	15.74
caseT07	910	115	16.71	15.68
caseT08	915	110	16.39	15.51
caseT09	920	105	16.00	15.38
caseT10	925	100	15.54	15.23
caseT11	930	95	14.96	15.04
caseT12	935	90	14.45	14.82
caseT13	940	85	13.76	14.53
caseT14	945	80	13.08	14.19
caseT15	950	75	12.24	13.84
T0704	930	95	14.97	15.04
T5115	870	104	15.85	15.35

## Maximum storm wave condition that influenced boulder distribution

Ideally, if there are several boulders with near equal dimension and weight, the maximum landward transport distances from the source should be about the same when topography and roughness on the reef can be assumed to be constants as like Kudaka Island. Moreover, the boulders could have been moved with the longer distance from the reef edge with decreasing boulder weight. Indeed, the distribution of the boulders with the longest distance from the reef edge among each weight shows the exponentially fining trend^16^. On the other hand, there are many boulders whose distribution distance is shorter than expected. Such boulders may have not been reached to the maximum possible distance yet because they have not been sufficiently affected by waves after their emplacements on the reef, although they still have chance to be transported further inland by forthcoming larger waves. Considering these distribution trends, each boulder with maximum inland distance should be adopted as the constraint of maximum wave estimation. Among the boulders with similar weights, the most landward ones are used as markers in this study. We divided boulders into seven groups by weight (Table 2) and adopted the landward boulders in each group as markers.

**Table 2:** The groups of boulders and information of each marker.

Group	Weight range (t)	Number	Marker’s distance (m)	Marker’s weight (t)
a	~2.5	101	275	0.6
b	2.5~5	49	264	3.6
c	5~10	39	229	8.5
d	10~20	15	191	17.7
e	20~50	4	106	24.4
f	50~100	1	95	54.3
g	100~	1	29	127.3

As shown in Fig. 3, there is a clear boundary whether all marker boulders are moved or not, suggesting that the boundary approximates wave conditions that can explain the present boulders’ distribution. It is important to note that the residual error, sum of the difference of transport distance of marker boulders, became minimum on the approximate curve (Fig. 3). If H_s_ is >20 m, residual error becomes significantly large irrespective of T_p_ (Fig. 3), because relatively small boulders were moved too much. On the other hand, when H_s_ is <9 m, some marker boulders could not be moved irrespective of T_p_, probably because the energy of the wave is dissipated on the reef and the wave force to move boulders cannot be maintained. Consequently, H_s_ ranging from 10 to 19 m would be the best explanation of the present distribution of boulders with <20 m in residual error.

Among the conditions that can move all marker boulders, the condition with the smallest residual error (13.32 m) is the wave condition with 13 m in H_s_ and 18 sec in T_p_. However, as seen in Fig. 3, a wave with a long T_p_ of 18 sec is difficult to be generated by the course of the typhoon set in our study. On the other hand, condition with the second smallest residual error (13.33 m) is the wave with 17 m and 16 sec. Therefore, this wave condition is more likely to be the maximum wave that can satisfy the present boulder distribution at Kudaka Island. Strictly speaking, the actual maximum wave conditions should be slightly smaller than the estimated condition. However, since the difference is sufficiently small (<1 m in wave height and <1 sec in period), the effect may be negligible in the discussion.

As stated above, the maximum significant wave observed at Nakagusuku Bay was 13.6 m in H_s_ and 14.9 sec in T_s_ during Typhoon 0704. Using Typhoon 0704 as an example, our simulation showed that the incident waves off Kudaka Island had H_s_ of 14.97 m and T_p_ of 15.04 sec. These values are still smaller than the estimated values of maximum waves (Fig. 3). Therefore, it is reasonable to conclude that the waves stronger than the maximum recorded storm wave around the Ryukyu Islands have hit to the study area in the past.

## Most intense typhoon in the northwestern Pacific Ocean

Since we independently estimated the wave conditions for arbitrary intensity bins of typhoons, it is also possible to estimate the maximum intensity of the typhoon that hit to the study area in the past. Interestingly, both curves estimated from two independent calculations (blue and red lines in Fig. 3) intersect at a point with wave conditions with H_s_ = 17.5 m and T_p_ = 15.9 sec; the central pressure is 890 hPa and the maximum wind speed is 135 knots. This in turn suggests that this size of typhoon can generate waves that carry enough energy to reproduce the present distribution of boulders. The condition at intersection (H_s_ = 17.5 m, T_p_ = 15.9 sec) is well consistent with the condition with low residual error estimated in the above section (H_s_ = 17 m, T_p_ = 16 sec). Therefore, we infer that this intensity of typhoon would be the maximum in the past since boulders were emplaced on the reef.

As can be seen from our calculation results, the strength of the typhoon and the magnitude of the waves are proportional, and so a small typhoon produces only small waves. Therefore, the cumulative effect of small but frequent typhoons did not affect the final boulder distribution. On the other hand, small typhoons may have a role of supplying new boulders onto the reef flat and slightly shift boulders landward.

In this study, we fixed the course of typhoon but one can consider the results might be different if we assume various courses. However, Okinawa Prefecture^31^ assumed similar course of typhoon as the possible maximum for risk assessment, suggesting that this course of typhoon can generate the largest storm wave. Also, even though we consider the other courses, the local wave condition at Kudaka Island should satisfy the wave conditions (red line) in Fig. 3. Therefore, typhoon intensity should not be extremely large irrespective of the course. Based on this evidence, we infer that our results likely approximated the maximum typhoon and storm waves irrespective of course of typhoon.

Regarding to the local risk assessment, Okinawa Prefecture^31^ assumed possible maximum typhoon with 870 hPa of the central pressure and 104 knots of the maximum wind speed. Although the assumption of central pressure is appropriate, the difference of maximum wind speed is too large to be ignored. Regarding to the wave condition, our simulation showed that H_s_ and T_p_ are 15.85 m and 15.35 sec, respectively, if we assume the model by Okinawa Prefecture^31^; it is slightly underestimated the calculated maximum wave from this study (Fig. 3). Based on these results, future update of supposition may be required for better risk assessment.

Then, how long can we go back in time to estimate maximum typhoon by storm wave boulders at Kudaka Island? We suggest that our results are applicable since at least 3500 years ago because of following reasons:

1. Morimoto *et al*.^32^ suggests that the sea surface temperature (SST) in summer has been maintained at 26 °C or more since about 7000 years ago at Kikai Island (Fig. 1), which is necessary SST to generate typhoon^33^.
2. Sea level fluctuation has been relatively stable since about 5000 years ago^34^.
3. Although it is necessary to estimate when the reef of Kudaka Island became similar to the present form, there is no information about the past reef formation at Kudaka Island. However, at Okinawa Island and Kume Island, which are close to Kudaka Island and located at similar latitude (Fig. 1), the formation and development of the reef topography have been investigated based on core drillings and radiocarbon dating^34,35^. According to the data, the fringing reef shape has not changed much over the past 4800 years at the southern area of Okinawa Island and the past 3500 years at Kume Island, respectively. Kawana^36^ also reported that the emplacement of boulders at Okinawa Island was started at least 3500 years ago. Similarly, displacement of boulders started 3000 years ago at Amami Islands^18^ and 4500 years ago at Ishigaki Island^26^ respectively.

For these reasons, it is reasonable to conclude that the present-style reef has changed little at Kudaka Island for at least 3500 years and the displacement of boulders might have started afterward. This is probably a conservative estimate; as the current reef crest around Kudaka Island was formed 4800 years ago^35^.

## Reconstructing intensities of tropical cyclones and corresponding waves

The maximum typhoon estimated in this study (890 hPa) was significantly stronger than the most powerful typhoon that hit to the Kudaka Island in the observation record from 1951 (930 hPa). However, Typhoon 6118, which passed to the east of the Ryukyu Islands and hit Honshu (Japan mainland) in 1961, had 145 knots of the maximum wind speed and 888 hPa of central pressure. It is as strong as the estimated typhoon, suggesting that present distribution of boulders at Kudaka Island can be explained if the equivalent size to the Typhoon 6118 pass near the island. The significant advantage of our method is that it can be applied anywhere in the world at places where storm wave boulders are reported. The boulders can be used to evaluate the intensity of tropical cyclones and the corresponding waves.

Our results suggest that the typhoon intensity was not extremely large in the past and there may be a certain physical limit to its development under stable climatic and oceanographic conditions. Meteorologically, there is a theory that typhoons cannot be abnormally large and the maximum intensity is constrained by the local environment^37–39^: typhoons have a certain limit, to which the can be developed physically (so-called Maximum Potential Intensity (MPI)). According to Emanuel^38^, MPI becomes stronger when 1) SST and boundary layer temperature are high, 2) the outflow temperature is low, and 3) the entropy difference on the sea surface is large. Since the boundary temperature and sea surface entropy are deeply related to SST, basically MPI strongly depends on SST. However, it was not unveiled whether the past typhoons before the observation era are within the range of MPI. It is not possible to estimate past MPI accurately without dense and accurate environmental values because it is difficult to calculate typhoon development completely even in current typhoon forecast^40^. In this sense, our methodology can estimate the maximum intensity of the past typhoons from the geological evidence. Since our result provides a realistic intensity (890 hPa) that is consistent with MPI, the MPI theory is supportable.

As stated above, we cannot fully exclude the effect of tsunamis unless we perform detail numerical analyses in the future work. However, the boulder distributions are almost identical throughout the entire Ryukyu Islands except for the tsunami-affected southern islands^18^. This sedimentological feature is reasonably explained by high-frequency but similar-size wave events by typhoons rather than low-frequency but different-size wave events (=tsunamis). Even if we assume that a tsunami had occurred in the past and formed the present boulder distribution at Kudaka Island, its size should be as large as the storm wave estimated in this study, which can be regarded as small. If we considering the potential effect of such minor tsunami effect, estimated wave and typhoon intensities in this study might have a chance of slight overestimation. However, the estimated intensities are close to the past ones such as the Typhoon 6118. Hence, we conclude that the estimated wave and typhoon intensities are reasonably valid as maximum.

## Methods

Numerical simulations of both (1) boulder transport by storm wave and (2) storm wave generation by arbitrary intensity of typhoon are carried out separately. Then, combining these two independent numerical results, the maximum intensities of waves and typhoons that formed present boulder distribution are estimated. The procedure is divided into two steps. Firstly, we estimate the maximum wave that has been affected to Kudaka Island based on the actual boulder distribution as a constraint. The computational domain is the coastal area of Kudaka Island, and the cross-sectional topographic data (5 m mesh) extracted from our multibeam bathymetric results^41^ was used for the modeling. Secondly, we estimate the intensity of a typhoon that can produces the maximum wave estimated in the first step. For this step, we use topographic data (30 seconds mesh) provided by GEBCO.

We conducted coupled calculation using two wave models, BOSZ (Boussinsq Ocean & Surf Zone model) and Delft3D-SWAN in order to express the non-hydrostatic motion in shallow waters and construct the wave and wind fields by the storm. BOSZ is a Boussinesq-type model that handles weakly dispersive waves, wave breaking, run-up on land and recirculation^42–44^. Note that this model includes the generation of infragravity waves and surf beat^45^. Calculation of the boulder movement is simultaneously performed with the wave-field calculation.

Some models of boulder movement have been proposed^13,46–48^. These models are commonly based on the Morison’s equation^49^ so that basic concept is similar. In this study, the equation of motion for boulder movement is based on Imamura *et al*.^13^ because the validity of this model was well confirmed by applying it to the 2004 Indian Ocean tsunami^50^. The external forces acting on the boulder are the fluid force *F*_*m*_, the friction force at the bottom *F*_*b*_, and the component of the gravity force *F*_*g*_ along the slope. According to Nandasena *et al*.^51^, sliding is the form with the smallest force required for movement. Also, on flat reef, boulder with low height are most likely to be moved by sliding^16^. Therefore, we assumed that the boulder is moved by sliding. We set the density of the boulder to 2.01, the coefficient of static friction to 0.75, and the coefficient of drag to 2.0 based on Goto *et al*.^16^. The coefficient of mass was used 1.67 based on Watanabe *et al*.^27^. The empirical dynamic friction proposed by Imamura *et al*.^13^ used for the simulation. This dynamic friction includes the effect of the lift force. We assumed that the roughness on the reef is constant for our calculation because the surface rock of the numerical region of boulder movement is composed of the same materials (reef rocks).

In order to calculate wave field caused by typhoon, we used Delft-3D^52^ and SWAN^53^. Delft-3D is a program to calculate the water elevation and current field by the shallow water equation. Receiving results from Delft-3D, then SWAN calculates the spectral parameters of the wave field including currents and storm surge; returning back the radiation stresses to Delft-3D.

The typhoon characteristics in the calculation are based on the method of Bricker *et al*.^54^. In order to calculate the pressure and wind fields, the path and central pressure of typhoon are input into the parametric model of Holland^55^. Then, a correction has been incorporated into the method based on Fujii and Mitsuta^56^ for considering asymmetry of those fields. The maximum wind radius was estimated using the empirical relationship of Quiring *et al*.^57^.

For the simulation using BOSZ, a set of H_s_ (24 cases) and T_p_ (23 cases) of wave spectrum are schanged independently, and the calculation is performed for 133 cases of initial wave conditions (Table S1). In order to reduce the computation load, we conducted one-dimensional calculation because topography, wave distribution, and boulder distribution don’t vary significantly in the north and south directions along the reef edge so that one dimensional calculations are representative for our purpose. The computation time is 3600 sec and the time step interval is around 0.05 sec (BOSZ uses an adaptive time stepping scheme). The water level is kept at mean tide level.

For Delft-3D/SWAN modeling, the course of typhoon is fixed as the path of Typhoon 0704, the course of which is considered to induce the largest effect of storm wave to the Kudaka Island. The calculations are performed under the conditions of 15 cases whose central pressure and the maximum wind speed are synchronously changed (Table 1). Also, Okinawa Prefecture^31^ conducted storm wave simulation for the generation of hazard map. They assume the typhoon with the central pressure of 870 hPa, with the maximum wind speed of 105 knot, and with a track going north on the west side of Okinawa Island similar to Typhoon 5115. Since the course of the simulation in this study is similar to the assumption by Okinawa prefecture^31^, it is possible to compare the results. Therefore, as shown as T5115 in Table 1, we simulated under the same condition as Okinawa prefecture^31^ to evaluate the validity of the damage estimation.

## Supplementary information

Supplementary information.

Supplementary Table S1.

Supplementary Figure S1.

Supplementary Figure S2.

Supplementary Figure S3.

Supplementary Figure S4.

Supplementary Figure S5.

## References

[1] Fritz HM (2007). Hurricane Katrina storm surge distribution and field observations on the Mississippi Barrier Islands. Estuar. Coast. Shelf Sci..

[2] Mas E (2015). Field survey report and satellite image interpretation of the 2013 Super Typhoon Haiyan in the Philippines. Nat. Hazards Earth Syst. Sci..

[3] Peduzzi P (2012). Global trends in tropical cyclone risk. Nat. Clim. Chang..

[4] Japan Meteorological Agency. Weather, Climate & Earthquake Information, http://www.jma.go.jp/jma/indexe.html (2019).

[5] Liu Kbiu, Shen C, Louie Ksheun (2001). A 1,000-year history of typhoon landfalls in Guangdong, Southern China, reconstructed from Chinese historical documentary records. Ann. Assoc. Am. Geogr..

[6] Switzer AD, Yu F, Gouramanis C, Soria JLA, Pham DT (2014). Integrating different records to assess coastal hazards at multi-century timescales. J. Coast. Res..

[7] Bateman MD (2018). Can sand dunes be used to study historic storm events?. Earth Surf. Process. Landforms.

[8] Swindles GT (2018). Sedimentary records of coastal storm surges: Evidence of the 1953 North Sea event. Mar. Geol..

[9] Emanuel K (2018). 100 Years of Progress in Tropical Cyclone Research. Meteorol. Monogr..

[10] Wallace EJ (2019). Intense Hurricane Activity Over the Past 1500 Years at South Andros Island, The Bahamas. Paleoceanogr. Paleoclimatology.

[11] Lau AYA (2016). Understanding the history of extreme wave events in the Tuamotu Archipelago of French Polynesia from large carbonate boulders on Makemo Atoll, with implications for future threats in the central South Pacific. Mar. Geol..

[12] Hongo C, Kurihara H, Golbuu Y (2018). Coral boulders on Melekeok reef in the Palau Islands: An indicator of wave activity associated with tropical cyclones. Mar. Geol..

[13] Imamura F, Goto K, Ohkubo S (2008). A numerical model for the transport of a boulder by tsunami. J. Geophys. Res. Ocean..

[14] Buckley ML, Wei Y, Jaffe BE, Watt SG (2012). Inverse modeling of velocities and inferred cause of overwash that emplaced inland fields of boulders at Anegada, British Virgin Islands. Nat. Hazards.

[15] Nandasena NAK, Paris R, Tanaka N (2011). Reassessment of hydrodynamic equations: Minimum flow velocity to initiate boulder transport by high energy events (storms, tsunamis). Mar. Geol..

[16] Goto K, Okada K, Imamura F (2009). Characteristics and hydrodynamics of boulders transported by storm waves at Kudaka Island, Japan. Mar. Geol..

[17] Kawana T, Nakata T (1994). Timing of Late Holocene Tsunamis Originated around the Southern Ryukyu Islands, Japan, Deduced from Coralline Tsunami Deposits. J. Geogr. Japan.

[18] Goto K, Miyagi K, Imamura F (2013). Localized tsunamigenic earthquakes inferred from preferential distribution of coastal boulders on the Ryukyu Islands, Japan. Geology.

[19] Seno T, Stein S, Gripp AE (1993). A model for the motion of the Philippine Sea Plate consistent with NUVEL-1 and geological data. J. Geophys. Res. Solid Earth.

[20] Ando M (2009). Is the Ryukyu subduction zone in Japan coupled or decoupled? —The necessity of seafloor crustal deformation observation. Earth Planets Sp..

[21] Goto K (2013). Re-evaluation of Hypocenter of the 1911 Great Earthquake around Kikai-jima, Japan. Zisin (Journal of the Seismological Society of Japan. 2nd ser.).

[22] Tadokoro K (2018). Interplate Coupling State at the Nansei-Shoto (Ryukyu) Trench, Japan, Deduced From Seafloor Crustal Deformation Measurements. Geophys. Res. Lett..

[23] Goto K, Kawana T, Imamura F (2010). Historical and geological evidence of boulders deposited by tsunamis, southern Ryukyu Islands, Japan. Earth-Science Rev..

[24] Nakamura M (2009). Fault model of the 1771 Yaeyama earthquake along the Ryukyu Trench estimated from the devastating tsunami. Geophys. Res. Lett..

[25] Okamura Y, Nishizawa A, Fujii Y, Yanagisawa H (2018). Accretionary prism collapse: a new hypothesis on the source of the 1771 giant tsunami in the Ryukyu Arc, SW Japan. Sci. Rep..

[26] Goto K, Miyagi K, Kawamata H, Imamura F (2010). Discrimination of boulders deposited by tsunamis and storm waves at Ishigaki Island, Japan. Mar. Geol..

[27] Watanabe M, Goto K, Imamura F, Hongo C (2016). Numerical identification of tsunami boulders and estimation of local tsunami size at Ibaruma reef of Ishigaki Island, Japan. Isl. Arc.

[28] Egashira, K., Fukuda, I., Kishira, Y. & Nishimura, T. Field measurement of the wave deformation on the reef. *Proc. Coastal Eng*., JSCE **32**, 90–94 (in Japanese) (1985).

[29] Ferrario F (2014). The effectiveness of coral reefs for coastal hazard risk reduction and adaptation. Nat. Commun..

[30] Kawaguchi, K., Suehiro, F., Fujiki, T. & Tamura, H. Annual Report on Nationwide Ocean Wave Information Network for Ports and Harbours (NOWPHAS 2016) (2018).

[31] Okinawa Prefecture. The investigation to estimate of tsunami and storm surge, (https://www.pref.okinawa.jp/kaigannbousai/con11/index.html) (2008).

[32] Morimoto M, Kayanne H, Abe O, McCulloch MT (2007). Intensified mid-Holocene Asian monsoon recorded in corals from Kikai Island, subtropical northwestern Pacific. Quat. Res..

[33] Palmén E (1948). On the Formation and Structure of Tropical Hurricanes. Geographica.

[34] Kan H, Takahashi T, Koba M (1991). Morpho-dynamics on Holocene reef accretion: drilling results from Nishimezaki Reef, Kume Island, the Central Ryukyus. Geographical Review of Japan.

[35] Kawana, T. Formative history of coral reef and earthquake and tsunami at Ryukyu Islands. *Ryukyu Islands Dur. Prehist. Historical**Age* 63–86 (in Japanese) (2011).

[36] Kawana T (2006). Invasion of about 3400 cal BP large wave in the southeastern Okinawa Island and the surroundings, the Ryukyus, Japan, as deduced from coralline deposits. Ryukyu Univ. kyouikugakubukiyo.

[37] Emanuel KA (1986). An Air-Sea Interaction Theory for Tropical Cyclones. Part I: Steady-State Maintenance. J. Atmos. Sci..

[38] Emanuel KA (1988). The Maximum Intencity of Hurricanes. J. Atmos. Sci..

[39] Emanuel KA (1995). Sensitivity of Tropical Cyclones to Surface Exchange Coefficients and a Revised Steady-State Model incorporating Eye Dynamics. J. Atmos. Sci..

[40] Ito K (2016). Errors in tropical cyclone intensity forecast by RSMC Tokyo and statistical correction using environmental parameters. Sci. Online Lett. Atmos..

[41] Minamidate, K. *et al*. Importance of high-resolution 3D topography for wave simulation. Proceedings of the 2019 International Conference on Climate Change, Disaster Management and Environmental Sustainability, 214–220 (2019).

[42] Roeber V, Cheung KF, Kobayashi MH (2010). Shock-capturing Boussinesq-type model for nearshore wave processes. Coast. Eng..

[43] Roeber V, Cheung KF (2012). Boussinesq-type model for energetic breaking waves in fringing reef environments. Coast. Eng..

[44] Li N, Yamazaki Y, Roeber V, Cheung KF, Chock G (2018). Probabilistic mapping of storm-induced coastal inundation for climate change adaptation. Coast. Eng..

[45] Roeber V, Bricker JD (2015). Destructive tsunami-like wave generated by surf beat over a coral reef during Typhoon Haiyan. Nat. Commun..

[46] Noji M (1993). Developing the method of tsunami boulder transport calculation. Coast. Eng. Japan Soc. Civ. Eng..

[47] Nandasena NAK, Tanaka N (2013). Boulder transport by high energy: Numerical model-fitting experimental observations. Ocean Eng..

[48] Kennedy AB (2016). Observations and Modeling of Coastal Boulder Transport and Loading during Super Typhoon Haiyan. Coast. Eng. J..

[49] O’Brien MP, Morison JR (1952). The forces exerted by waves on objects. Trans. - Am. Geophys. Union.

[50] Goto K, Okada K, Imamura F (2010). Numerical analysis of boulder transport by the 2004 Indian Ocean tsunami at Pakarang Cape, Thailand. Mar. Geol..

[51] Nandasena NAK, Paris R, Tanaka N (2011). Numerical assessment of boulder transport by the 2004 Indian ocean tsunami in Lhok Nga, West Banda Aceh (Sumatra, Indonesia). Comput. Geosci..

[52] Deltares. Delft3D 3D-FLOW user manual. 3.15.34158 (2014).

[53] Booij N, Ris RC, Holthuijesen LH (1999). A third-generation wave model for coastal regions 1. Model description and validation. J. Geophys. Res. Ocean..

[54] Bricker JD (2014). Spatial Variation of Damage due to Storm Surge and Waves during Typhoon Haiyan in the Philippines. J. Japan Soc. Civ. Eng..

[55] Holland GJ (1980). An Analytic Model of the Wind and Pressure Profiles in Hurricanes. Mon. Weather Rev..

[56] Fujii T, Mitsuta Y (1986). Synthesis of a Stochastic Typhoon Model and Simulation of Typhoon Winds. Annuals Disaster Prevention Research Institute, Kyoto University..

[57] Quiring S, Schumacher A, Labosier C, Zhu L (2011). Variations in mean annual tropical cyclone size in the Atlantic. J. Geophys. Res. Atmos..

