# Supplementary material for: Millennial scale maximum intensities of typhoon and storm wave in the northwestern Pacific Ocean inferred from storm deposited reef boulders
Source: Sci Rep. 2020 Apr 29;10:7218. doi: 10.1038/s41598-020-64100-6 (PMC7190612; doi:10.1038/s41598-020-64100-6)
Supplement: Supplementary file 1 — Supplementary information. [file 41598_2020_64100_MOESM1_ESM.docx]

Supplementary Information for

**Millennial scale maximum intensities of typhoon and storm wave**

**in the northwestern Pacific Ocean inferred from storm deposited reef boulders**

Kenta Minamidate*, Kazuhisa Goto, Masashi Watanabe, Volker Roeber, Ken Toguchi,

Masami Sannoh, Yosuke Nakashima, Hironobu Kan

*Correspondence to: kenta.minamidate.t7@dc.tohoku.ac.jp

Supplementary file captions

Figure S1. Conceptual models showing (a) initial boulder distribution, and during (b) small storm, (c) large storm, (d) small tsunami, and (e) large tsunami together with (f) current boulder distribution in Kudaka Island. When small storm waves (b) and tsunamis (d) affected, some small boulders may be emplaced and moved landward but certain weight of boulders deposited in the maximum landward distances from the reef edge wouldn’t be moved by these events. Large tsunamis (e) have significant wave force to move boulders further inland so that it is inconsistent with the field observation at Kudaka Island. Therefore, current boulder distribution can be interpreted to have been controlled only by the most intense storm wave.

Figure S2. The time series variation of wave calculation in caseB04 (see Table S1). Waves of O, P, Q and R move and deform with time. The waveform of O (offshore wave) is almost constant regardless of time. However, the waveforms of P and Q are shorter in wavelength and higher in height as they go to shallow sea. The wave height further increases near the seaward-side of the reef edge as wave R shows, then it breaks at the reef edge.

Figure S3. The distribution of maximum water level and maximum water velocity in caseB04 (upper figure) and topography (lower figure).

Figure S4. The initial and final positions of the four largest boulders in caseB04 (see Table S1).

Figure S5. Results of storm simulation for (a) caseT02 ~ (n) caseT15.

Table S1. All initial wave conditions and results of boulder transport.
