# Supplementary figures and images for: Millennial scale maximum intensities of typhoon and storm wave in the northwestern Pacific Ocean inferred from storm deposited reef boulders
Source: Sci Rep. 2020 Apr 29;10:7218. doi: 10.1038/s41598-020-64100-6 (PMC7190612; doi:10.1038/s41598-020-64100-6)

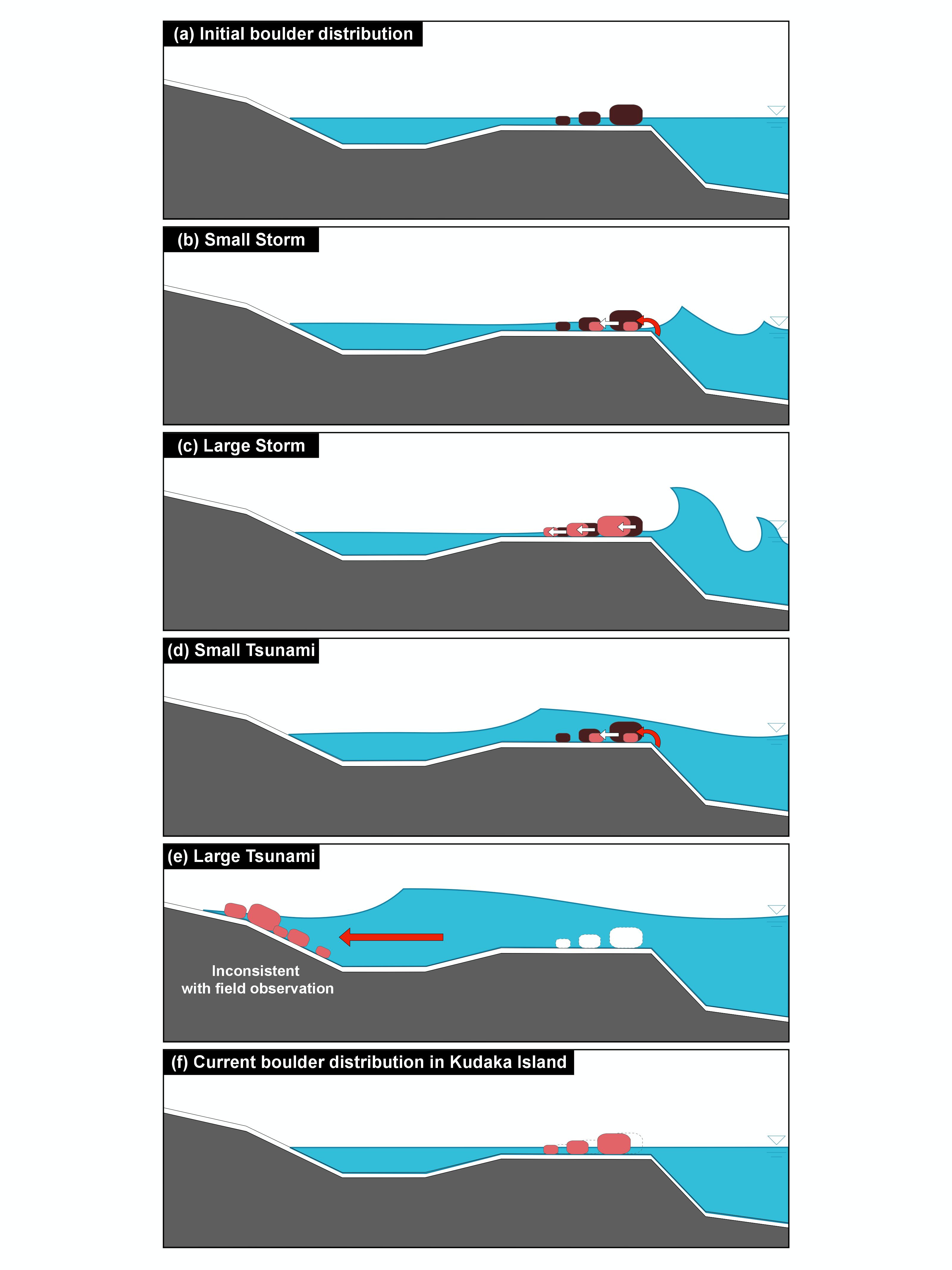

Supplement: Supplementary file 3 — Supplementary Figure S1. [file 41598_2020_64100_MOESM3_ESM.jpg]

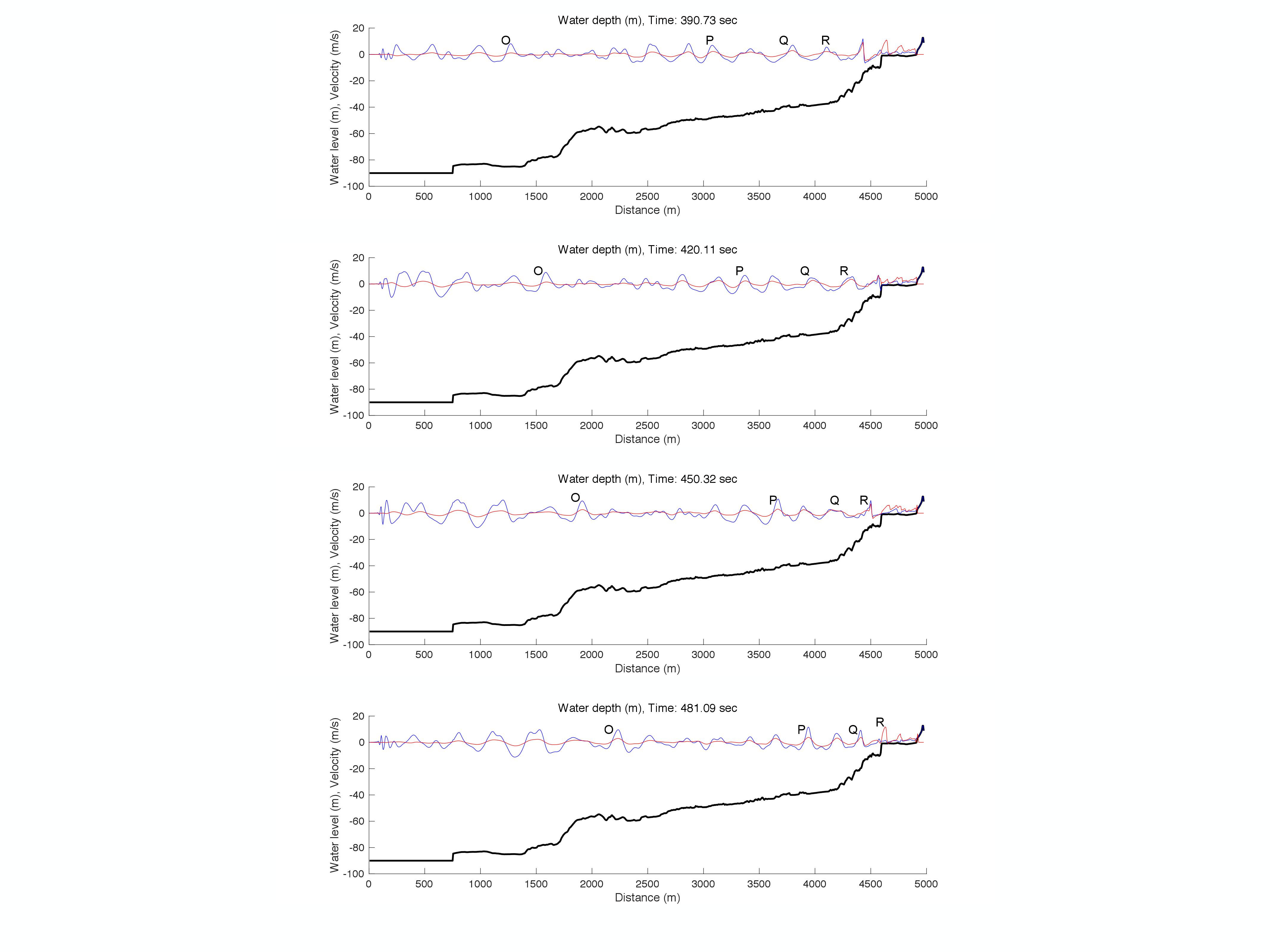

Supplement: Supplementary file 4 — Supplementary Figure S2. [file 41598_2020_64100_MOESM4_ESM.jpg]

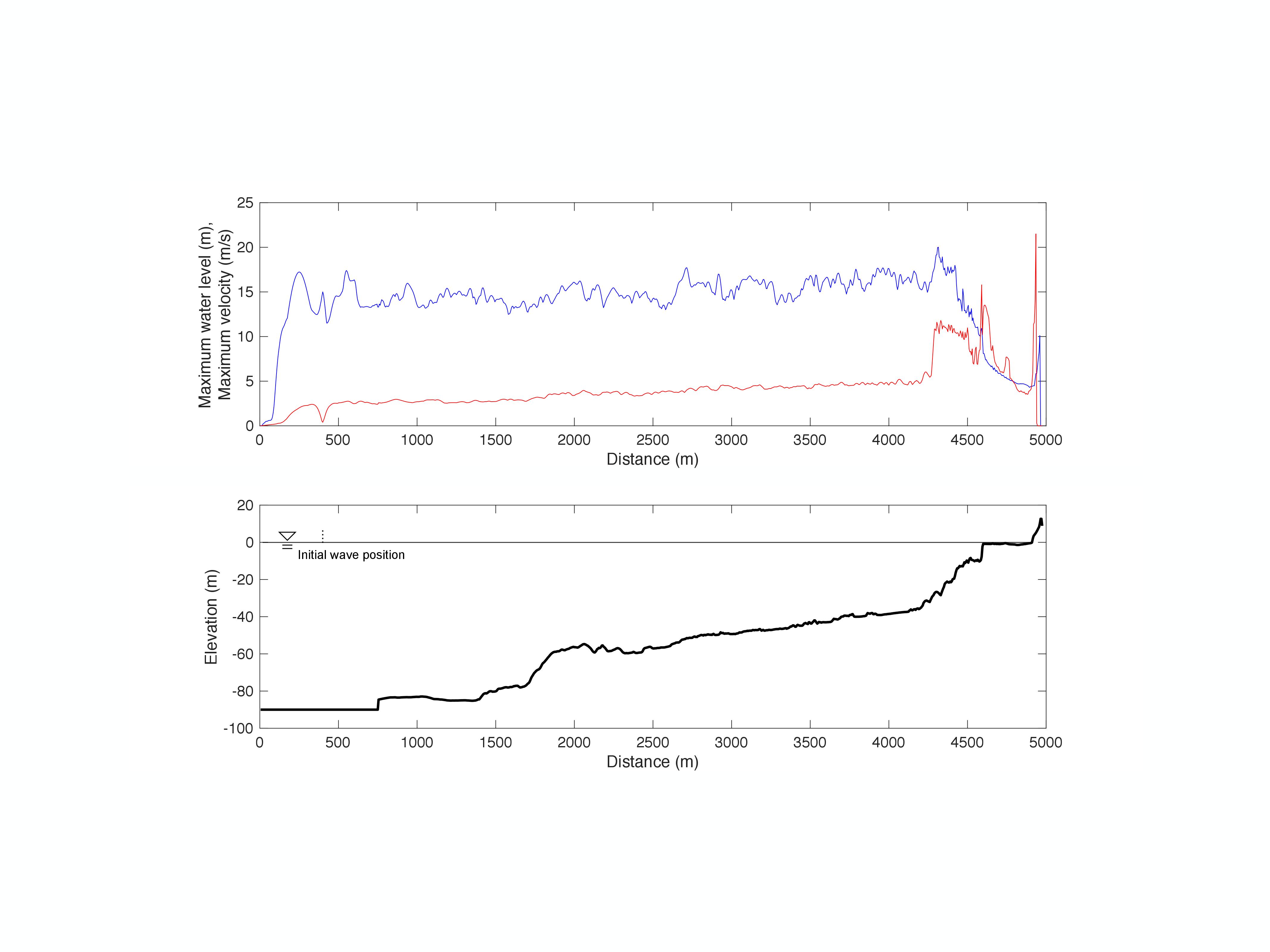

Supplement: Supplementary file 5 — Supplementary Figure S3. [file 41598_2020_64100_MOESM5_ESM.jpg]

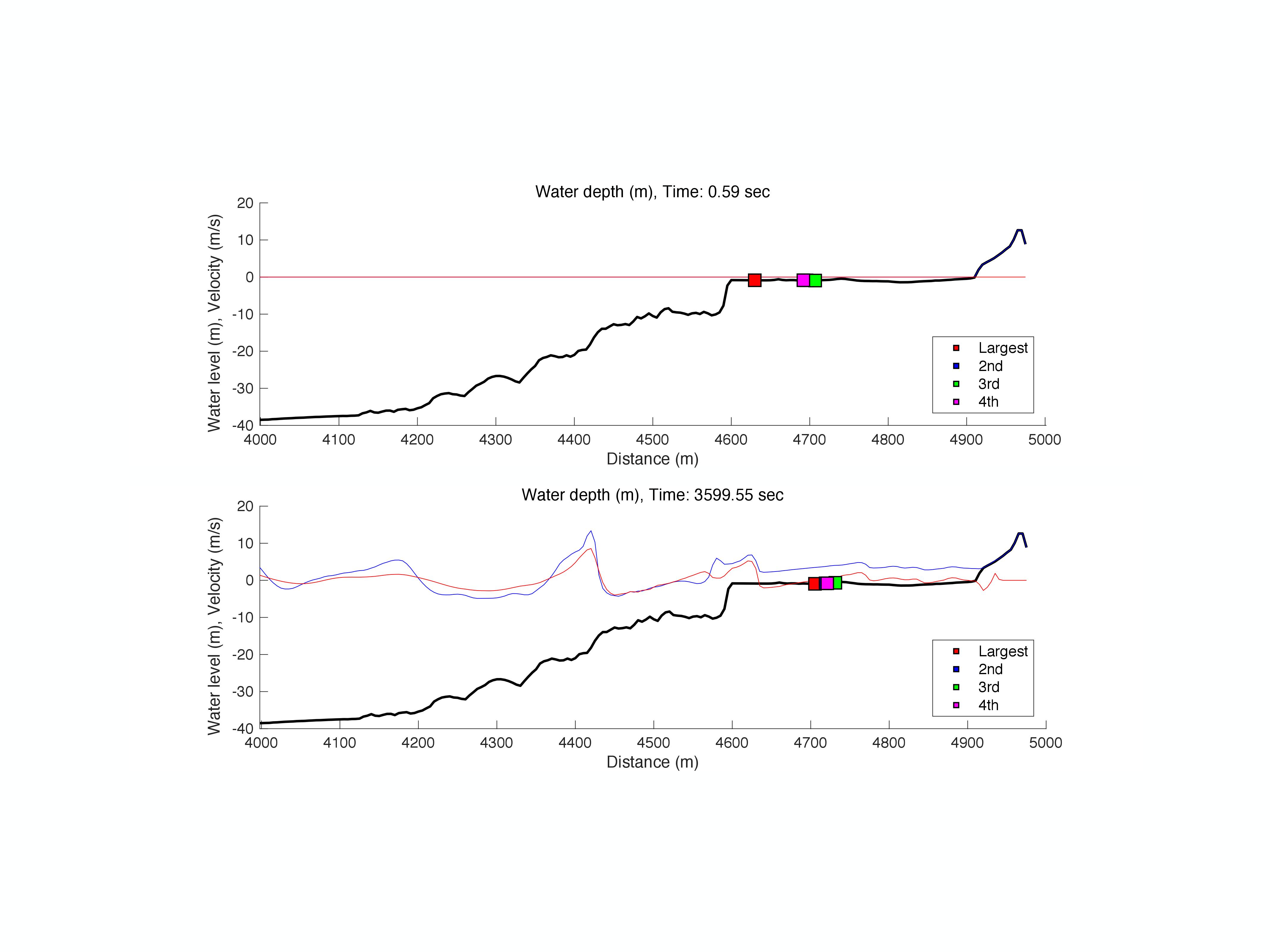

Supplement: Supplementary file 6 — Supplementary Figure S4. [file 41598_2020_64100_MOESM6_ESM.jpg]

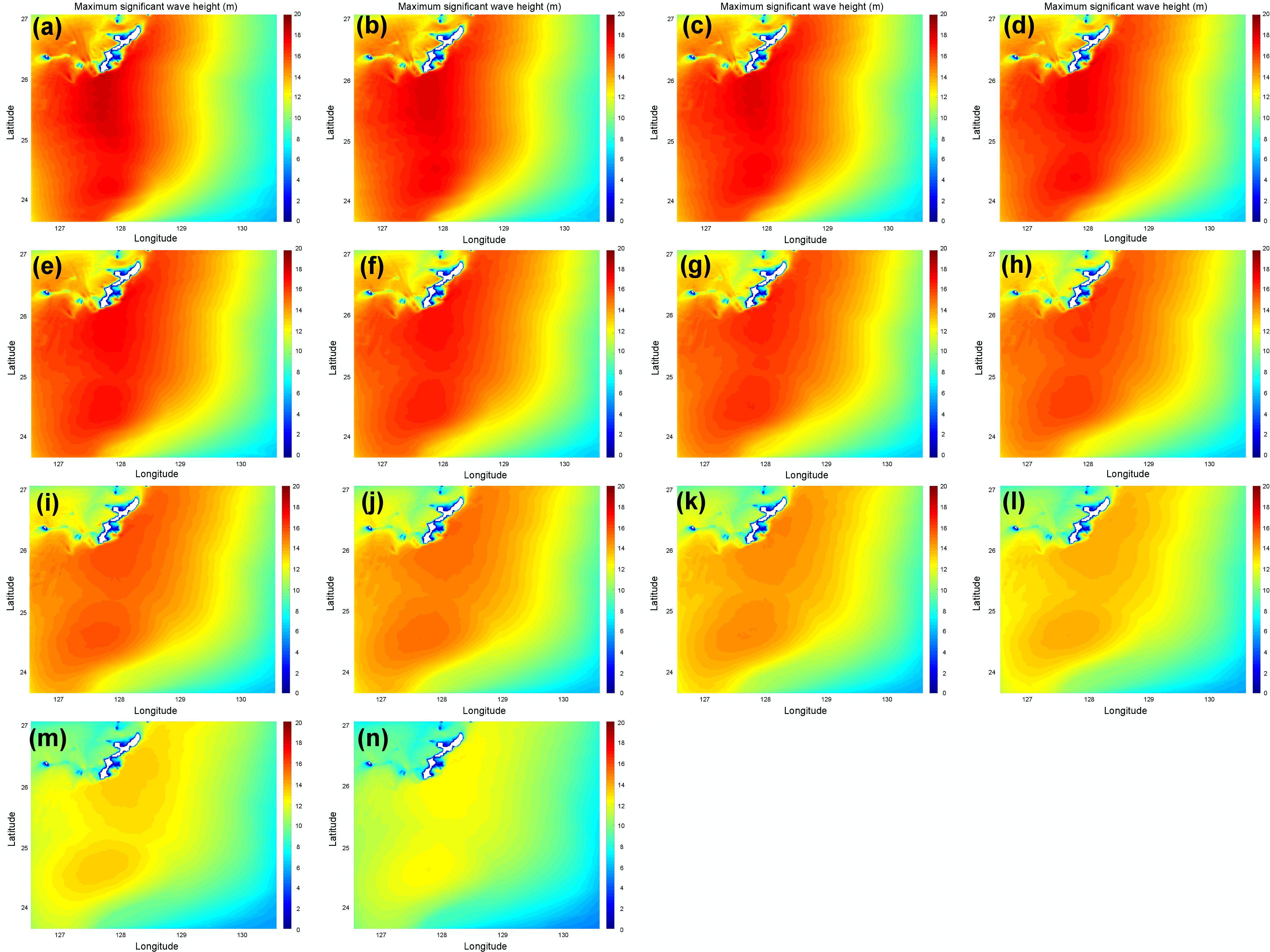

Supplement: Supplementary file 7 — Supplementary Figure S5. [file 41598_2020_64100_MOESM7_ESM.jpg]
